# Survey of the use of ancillary tests in the diagnosis of brain stem death

**DOI:** 10.1186/cc9943

**Published:** 2011-03-11

**Authors:** A Sherrington, L Barrass, G Mandersloot, M Healy

**Affiliations:** 1 The Royal London, London, UK

## Introduction

In the UK, the diagnosis of brain stem death (BSD) is mainly confirmed by clinical testing. The Code of Practice guidelines regarding the time interval between cessation of sedative drugs and testing allow for considerable variation in interpretation and practice [1]. In some countries, ancillary tests are used as an alternative to clinical diagnosis [2]. Our aim was to survey current attitudes and practice surrounding the use of ancillary tests in the diagnosis of BSD.

## Methods

We confirmed ethics committee exemption and the survey was peer-reviewed by the Neuro-critical Care Network. We distributed it electronically to the 31 neuro-critical care centres in the UK, collecting responses anonymously.

## Results

We had a response rate of 94%. The majority of centres had four-vessel angiography (4VA) and spiral CT angiography (CTA) available (25 and 24, respectively), 13 centres had access to transcranial Doppler (TCD) and 12 to evoked potentials (EPs). Twenty-three centres had used or would consider ancillary tests in addition to clinical tests where these could not be completed due to the nature of injury (for example, facial trauma); 19 centres had used or would consider this when drug levels were unavailable. Four centres had used ancillary tests alone to confirm BSD where depressant drug levels had precluded clinical testing; a further eight centres would consider this approach. First-choice investigation was CTA in 48% of centres, 30% preferred 4VA, and 4% TCD. Following cessation of sedative drugs, there was considerable variation in timing of clinical testing and pharmacokinetic factors considered. Whilst 33% of centres measure thiopentone levels in all cases, 22% never do. In five centres, delays in testing due to raised drug levels exceeded 5 days. Where raised levels preclude testing, 17 centres were confident that 4VA would diagnose BSD in some or all circumstances; 13 centres were confident with CTA. TCD and EPs were considered less reliable.

## Conclusions

There is considerable variation in UK opinion and practice. Measurement of drug levels is not universal but raised levels delay diagnosis significantly. More than one-half of the centres surveyed would be confident in using an ancillary test alone to diagnose BSD. Further consensus is needed.
